# Apixaban vs. Warfarin in Atrial Fibrillation Patients With Chronic Kidney Disease

**DOI:** 10.3389/fcvm.2021.752468

**Published:** 2021-10-18

**Authors:** Chung-Ming Fu, Lung-Chih Li, Yueh-Ting Lee, Shih-Wei Wang, Chien-Ning Hsu

**Affiliations:** ^1^Division of Nephrology, Department of Internal Medicine, Kaohsiung Chang Gung Memorial Hospital, Chang Gung University College of Medicine, Kaohsiung, Taiwan; ^2^Institute for Translational Research in Biomedicine, Kaohsiung Chang Gung Memorial Hospital, Chang Gung University College of Medicine, Kaohsiung, Taiwan; ^3^Department of Pharmacy, Kaohsiung Chang Gung Memorial Hospital, Kaohsiung, Taiwan; ^4^School of Pharmacy, Kaohsiung Medical University, Kaohsiung, Taiwan

**Keywords:** apixaban, warfarin, chronic kidney disease, atrial fibrillation (AF), ischemic stroke, bleeding, thromboembolism

## Abstract

**Background and Objectives:** Real-world evidence of apixaban treatment in patients with chronic kidney disease remains scarce. This study aimed to compare the relative risk of stroke or systemic embolism (SE) and major bleeding between apixaban and warfarin in atrial fibrillation (AF) patients with different degrees of kidney function.

**Design, Setting, Participants, and Measurements:** We evaluated newly diagnosed AF patients between 2004 and 2018, who were receiving apixaban or warfarin. Electronic medical record data were collected from a large healthcare delivery network in Taiwan. The outcomes of hospitalization for stroke/SE and major bleeding were compared with propensity-score matched apixaban and warfarin cohorts. Stratified analyses according to initial apixaban dose (standard dose of 10 mg/day vs. lower dose of 2.5–5.0 mg/day) and baseline estimated glomerular filtration rate were performed.

**Results:** Each cohort involved 1,625 matched patients. Apixaban was significantly associated with a lower risk of stroke/SE (adjusted hazard ratio [aHR]: 0.74; 95% confidence interval [CI]:0.57–0.97; *p* = 0.03). The risk of major bleeding was not increased whether in standard doses (aHR: 0.66; 95% CI: 0.45–0.96; *p* = 0.03) or reduced doses (aHR, 0.84; 95% CI, 0.63–1.12; *p* = 0.23) of apixaban. Regarding kidney function, apixaban reduced the risk of stroke/SE by 37% in those with an eGFR of <30 ml/min/1.73 m^2^ (aHR: 0.63; 95% CI: 0.40–0.98; *p* = 0.04).

**Conclusions:** Compared to warfarin, apixaban is associated with a reduced risk of stroke/SE and is consistent with a subset of AF patients with eGFR <30 ml/min/1.73 m^2^. Both standard and reduced doses of apixaban showed lower risk of major bleeding than those of warfarin.

## Introduction

Atrial fibrillation (AF) as the most common cardiac arrhythmia (1) and contributes significantly to cerebral ischemic stroke and other severe thromboembolic events. To prevent these severe complications, current guidelines stipulate that high-risk AF patients (CHA_2_DS_2−_VASc scores ≥2) should be prescribed direct oral anticoagulants (DOACs) rather than vitamin K antagonists (2–5). Patients with chronic kidney disease (CKD) have a 2- to 3-fold higher prevalence of AF than the general population (6–8). In addition, CKD itself contributes to a pro-thrombotic state, which increases the risks of ischemic stroke or systemic embolism (9–11). The risk of thromboembolic events is even worse in CKD patients receiving renal replacement therapy (11, 12). Furthermore, patients with an estimated glomerular filtration rate (eGFR) of <30 mL/min/1.73 m^2^ have a higher risk of bleeding compared to those with an eGFR of between 30 and 60 mL/min/1.73 m^2^ and those with an eGFR ≥ 60 mL/min/1.73 m^2^ while receiving oral anticoagulant (OAC) therapy (12–14). Importantly, most pivotal studies of DOACs excluded patients with advanced CKD and end-stage kidney disease (ESKD). Thus, real-world evidence is needed to optimize the prevention of thromboembolism, and still minimize the risk of bleeding in patients with abnormal kidney function.

A patient's kidney function is one of the factors that influences OAC selection (15, 16), and warfarin is often prescribed in patients with CKD. Apixaban is currently the only approved DOAC for AF patients with serum creatinine clearance (CrCl) of <15 mL/min; however, approval was based on a pharmacokinetic study of only eight patients with CKD on dialysis (17). Furthermore, treatment outcomes of apixaban in ESKD patients have been reported (18, 19). A study using 2010–2015 Renal Data System in the United State (USRDS) data found that although apixaban has no benefit on stroke/systemic embolism (SE) prevention, it is associated with a significantly lower risk of major bleeding compared to warfarin (18). Another study using USRDS data (2012–2015) compared apixaban with no anticoagulation in patients with chronic dialysis and AF, and found that apixaban treatment was not associated with risk reductions in both ischemic stroke and fatal or intracranial bleeding (19). Given that these studies mainly focused on the necessity of anticoagulation in the chronic dialysis population, the usefulness of apixaban treatment in CKD patients without dialysis treatment is still unclear. Thus, this study aimed to compare the relative risk of stroke or SE and major bleeding between apixaban and warfarin in AF patients with different degrees of kidney disease.

## Materials and Methods

### Study Design and Data Source

This was a retrospective cohort study of adult patients with non-valvular AF or atrial flutter. Data were obtained from the Chang Gung Research Database (CGRD), a de-identified, electronic health records database of patient information from the healthcare delivery system in Taiwan. The CGRD contains International Classification of Diseases, Ninth/Tenth Revision, Clinical Modification *(*ICD-9/10-CM*)* codes, Healthcare Common Procedure Coding System codes, Anatomical Therapeutic Chemical Classification System codes, and laboratory test results in emergency departments and in-and-outpatient settings (Supplementary Table 1). The data sets and have been described previously (20, 21).

This study was approved by the Institutional Review Board of Chang Gung Medical Foundation at Taipei, Taiwan (approval number 201900901B0) and was conducted according to the tenets of the Declaration of Helsinki. The need for informed consent was waived owing to the retrospective nature of the study.

### Patients

**Figure 1:**
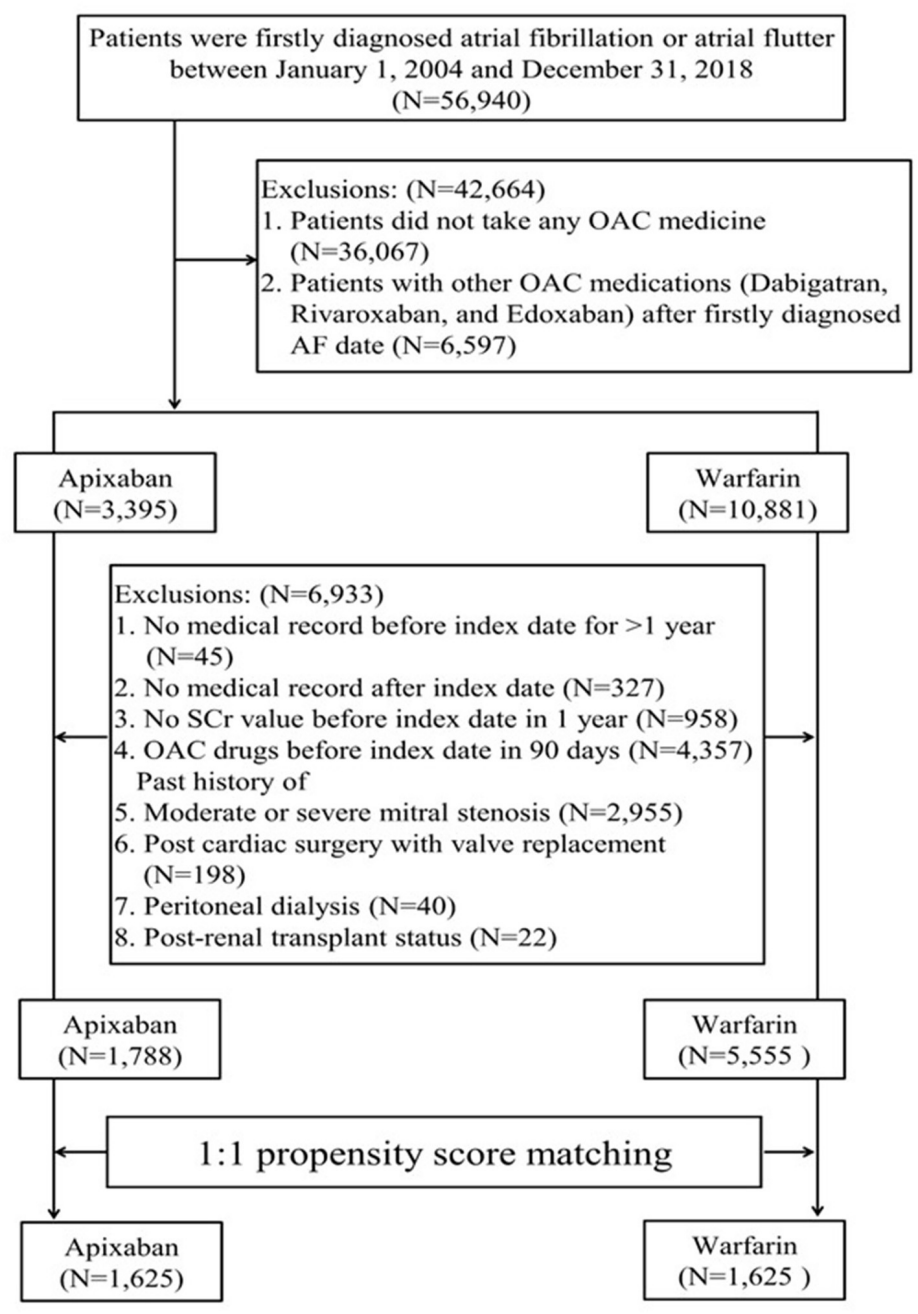
Patient inclusion flowchart.

We evaluated AF patients who were newly diagnosed between January 1, 2004, and December 21, 2018, in whom apixaban or warfarin therapy had been initiated. The inclusion criteria were having a diagnosis of AF before the index date (the date of apixaban or warfarin initiation) and at least one or more admissions within at least 12 months before the index date (Figure 1). Patients were excluded if they had any of the following: OAC treatment (warfarin, apixaban, dabigatran, rivaroxaban, edoxaban) within 3 months before the index date, missing serum creatinine (SCr) results, moderate or severe mitral stenosis, valve replacement, peritoneal dialysis, or kidney transplantation. The patient selection criteria are detailed in Figure 1, Supplementary Table 1. The patients were identified using ICD-9/10-CM codes on at least two outpatient visits with an interval of more than 28 days or on one post-discharge follow-up within the study period. The first apixaban or warfarin prescription date in the outpatient setting was designated as the index date for patients without any other OAC treatment.

### Outcome Measures

Effectiveness was evaluated according to the incidence of stroke or SE as outcome measure, while safety was evaluated according to the incidence of major bleeding, including any intracranial hemorrhage but not traumatic hemorrhage, intraabdominal, gastrointestinal bleeding, hematuria, or bleeding at other sites (Supplementary Table 1) (22). The outcomes of interest were defined according to discharge diagnosis in the as-treated cohort. All patients were followed up from the index date to the first event of interest, discontinuation date of apixaban or warfarin, medication switch date, in-hospital death, loss to follow-up (≥365 days without any medical encounters before the end date of the database), or the data cut-off date (December 31, 2018), whichever came first.

### Statistical Analysis

The patients were matched using propensity scores (PS) to minimize selection bias (23, 24). Each patient's PS was calculated based on the following characteristics: demographic data, such as age and sex; individual disease condition in the Charlson Comorbidity Index (25); eGFR; hypertension; major bleeding; medications; CHA_2_DS_2_-VASc score and HAS-BLED score. The covariates for the PS matching model are presented in Table 1, Supplementary Table 1. Patients who were prescribed apixaban or warfarin were matched in a 1:1 ratio using a greedy algorithm (26). The distributions of PS was assessed, and the covariate balance between treatment groups were determined using the standardized mean differences (SMD) with a threshold of <10% (27). The relative risk of stroke/SE and major bleeding between apixaban and warfarin was evaluated using Cox proportional hazards models separately, and adjusted for covariates listed in Table 1.

**Table 1:** Baseline patient characteristics by treatment group before and after propensity score matching.

	**Before propensity score matching**			**After propensity score matching**		
	**Warfarin (*n* = 5,555)**	**Apixaban (*n* = 1,788)**	**SMD**	**Warfarin (*n* = 1,625)**	**Apixaban (*n* = 1,625)**	**SMD**
**Age group, years**, ***n*** **(%)**						
<40	122 (2.20)	12 (0.67)	0.129	11 (0.68)	12 (0.74)	0.007
40–64	1,882 (33.88)	250 (13.98)	0.480	257 (15.82)	245 (15.08)	0.020
65–74	1,578 (28.41)	537 (30.03)	0.036	503 (30.95)	504 (31.02)	0.001
≥75	1,973 (35.52)	989 (55.31)	0.406	854 (52.55)	864 (53.17)	0.012
**Sex**, ***n*** **(%)**						
Male	3,335 (60.04)	1,023 (57.21)	0.057	926 (56.98)	938 (57.72)	0.015
Female	2,220 (39.96)	765 (42.79)	0.057	699 (43.02)	687 (42.28)	0.015
**Baseline eGFR, ml/min/1.73 m**^**2**^, ***n*** **(%)**						
≧90	1,001 (18.02)	286 (16.00)	0.054	286 (17.60)	277 (17.05)	0.015
60–89.9	2,262 (40.72)	739 (41.33)	0.012	678 (41.72)	674 (41.48)	0.005
45–59.9	1,066 (19.19)	386 (21.59)	0.060	337 (20.74)	357 (21.97)	0.030
30–44.9	617 (11.11)	246 (13.76)	0.080	193 (11.88)	198 (12.18)	0.010
15–29.9	298 (5.36)	115 (6.43)	0.045	117 (7.20)	103 (6.34)	0.034
<15	311 (5.60)	16 (0.89)	0.268	14 (0.86)	16 (0.98)	0.013
**Charlson comorbid conditions**, ***n*** **(%)**						
Acute myocardial infarction	354 (6.37)	103 (5.76)	0.026	94 (5.78)	95 (5.85)	0.003
Congestive heart failure	1,857 (33.43)	568 (31.77)	0.036	537 (33.05)	519 (31.94)	0.024
Peripheral vascular diseases	245 (4.41)	44 (2.46)	0.107	29 (1.78)	44 (2.71)	0.062
Cerebral vascular accident	2,013 (36.24)	589 (32.94)	0.069	505 (31.08)	525 (32.31)	0.026
Dementia	135 (2.43)	51 (2.85)	0.026	49 (3.02)	48 (2.95)	0.004
Pulmonary disease	1,031 (18.56)	291 (16.28)	0.060	261 (16.06)	277 (17.05)	0.027
Connective tissue disorder	26 (0.47)	17 (0.95)	0.058	14 (0.86)	14 (0.86)	0.000
Peptic ulcer	766 (13.79)	291 (16.28)	0.070	270 (16.62)	266 (16.37)	0.007
Liver diseases	553 (9.95)	120 (6.71)	0.118	117 (7.20)	116 (7.14)	0.002
Diabetes	1,563 (28.14)	537 (30.03)	0.042	470 (28.92)	477 (29.35)	0.010
Diabetes with complications	375 (6.75)	193 (10.79)	0.143	145 (8.92)	143 (8.80)	0.004
Paraplegia	249 (4.48)	98 (5.48)	0.046	87 (5.35)	82 (5.05)	0.014
Renal disease	762 (13.72)	424 (23.71)	0.258	311 (19.14)	316 (19.45)	0.008
Cancer	340 (6.12)	187 (10.46)	0.158	147 (9.05)	151 (9.29)	0.009
Severe liver diseases	18 (0.32)	6 (0.34)	0.002	6 (0.37)	5 (0.31)	0.011
Metastatic cancer	59 (1.06)	30 (1.68)	0.053	25 (1.54)	28 (1.72)	0.015
Hypertension	3,612 (65.02)	1,314 (73.49)	0.184	1,186 (72.98)	1,177 (72.43)	0.012
Prior major bleeding	1,147 (20.65)	582 (32.55)	0.272	479 (29.48)	484 (29.78)	0.007
**Prior medication uses**						
Lipid-lowering agent	905 (16.29)	485 (27.13)	0.265	409 (25.17)	408 (25.11)	0.001
Glucose-lowering agent	1,088 (19.59)	403 (22.54)	0.073	342 (21.05)	340 (20.92)	0.003
Anti-hypertension	3,857 (69.43)	1,394 (77.96)	0.195	1,262 (77.66)	1,246 (76.68)	0.024
Anti-platelet agent	2,433 (43.80)	753 (42.11)	0.034	708 (43.57)	690 (42.46)	0.022
Aspirin	2,029 (36.53)	560 (31.32)	<0.0001	583 (35.88)	512 (31.51)	-
Clopidogrel	552 (9.94)	261 (14.6)	<0.0001	171 (10.52)	238 (14.65)	-
Ticagrelor	12 (0.22)	14 (0.78)	<0.0001	3 (0.18)	11 (0.68)	-
Others	264 (4.75)	67 (3.75)	0.112	72 (4.43)	60 (3.69)	-
Amiodarone	841 (15.14)	355 (19.85)	0.124	302 (18.58)	301 (18.52)	0.002
Digoxin	839 (15.10)	150 (8.39)	0.210	149 (9.17)	146 (8.98)	0.006
NSAIDs	568 (10.23)	200 (11.19)	0.031	189 (11.63)	185 (11.38)	0.008
Gastric antacids	922 (16.60)	506 (28.30)	0.283	421 (25.91)	423 (26.03)	0.003
**Mean value (SD)**						
CHA_2_DS_2_-VASc score	3.40 (1.84)	3.92 (1.70)	0.293	3.81 (1.69)	3.83 (1.68)	0.011
HAS-BLED score	2.55 (1.43)	3.02 (1.36)	0.333	2.92 (1.36)	2.92 (1.34)	0.003

*SMD, standardized mean difference; SD, standard deviation; CKD, chronic kidney disease; eGFR, estimated glomerular filtration rate; NSAID: non-steroidal anti-inflammatory drug*.

Subgroup analysis according to the apixaban dose (standard dose [10 mg/day] vs. reduced dose [2.5–5.0 mg/day]) was conducted using the Cox proportional hazards model to evaluate the dose relationship with the heterogeneity of treatment effects. Subgroup analysis according to baseline eGFR (≥60 ml/min/1.73 m^2^ (mild CKD), 59.9–30.0 ml/min/1.73 m^2^ (moderate CKD), and <30 ml/min/1.73 m^2^ (advanced CKD) was also performed to evaluate the influence of kidney function on the effectiveness of apixaban and warfarin. Baseline eGFR was calculated based on the mean serum creatinine (SCr) level within 3 months prior to the index date and using the Modification of Diet in Renal Disease (MDRD) equation (28): 175 × SCr (mg/dL)^−1.154^ × age (years)^−0.203^ × 0.742 (if female). Furthermore, hospital admissions for pneumonia or hip fracture were regarded as negative control outcomes (19) to ensure the robustness of the study results. We hypothesized that pneumonia and hip fracture had the same exposure risk in the apixaban and warfarin groups. All statistical analyses were performed using SAS 4.0 (Cary, NC, USA). A two-sided *P*-value of < 0.05 was considered statistically significant.

## Results

### Patient Characteristics

A total of 56,940 patients with AF or atrial flutter diagnosis were identified. Out of them, 7,343 patients who were administered warfarin (*n* = 5555) or apixaban (*n* = 1788) were initially evaluated (Figure 1). Before matching, the apixaban group were more likely to be older (mean age: 75.16 ± 10.63 years vs. 68.72 ± 12.47 years) at the index date and had higher CHA_2_DS_2_-VASc (3.92 ± 1.70 vs. 3.4 ± 1.84) and HAS-BLED (3.02 ± 1.36 vs. 2.55 ± 1.43) scores. Further, comorbid kidney disease was more prevalent in the apixaban group than in the warfarin group (23.71 vs. 13.72%). However, the baseline mean eGFR was similar between the two groups.

After matching, the PS distributions were compatible and baseline characteristics were similar in the matched cohort, with each group involving 1,625 patients. The SMDs of all variables were <0.1 (Table 1). The mean age at the initiation of apixaban or warfarin was 74–75 years. In total, 131 patients (8.06%) in the warfarin group and 119 patients (7.32%) in the apixaban group had advanced CKD (i.e., eGFR <30 ml/min/1.73 m^2^). Among the patients who received apixaban, 710 patients (56.31%) and 913 (43.69%) patients received a reduced and standard dose, respectively. The reduced and standard dose subgroups had a mean age of 78.5 years and 70 years, respectively (Supplementary Table 2). The patient characteristics before and after PS matching are shown in Table 1.

### Study Outcomes

**Figure 2:**
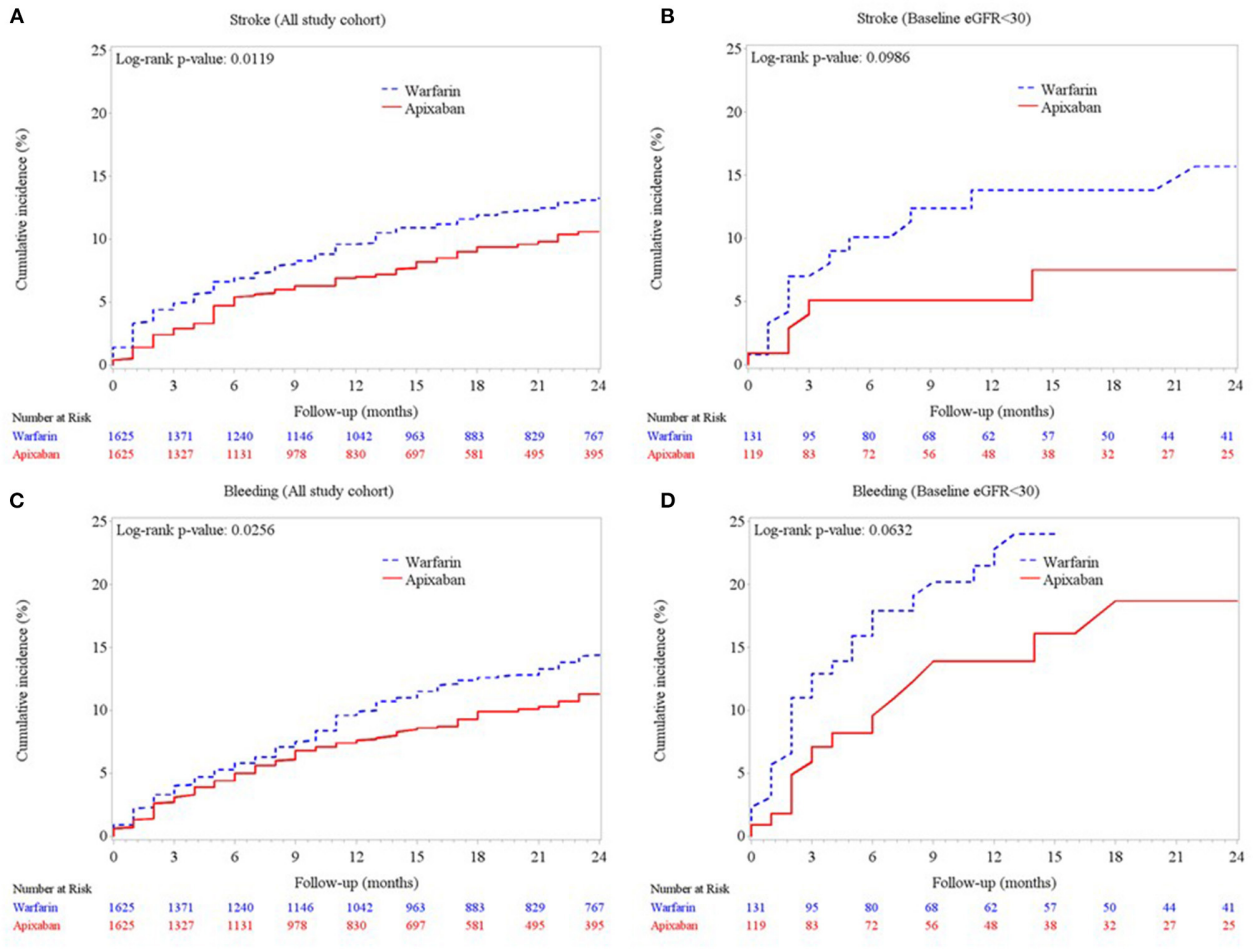
Cumulative incidences of **(A)** ischemic stroke/systemic embolism in the matched cohort, **(B)** ischemic stroke/systemic embolism in the subgroup of patients with advanced CKD (eGFR <30 ml/min/1.73 m^2^), **(C)** major bleeding in the matched cohort, and **(D)** major bleeding in the subgroup of patients with eGFR <30 ml/min/1.73 m^2^.

The rates of stroke/SE, major bleeding, and in-hospital mortality are presented in Table 2. Compared to the warfarin group, the apixaban group showed significantly lower incidence rates of stroke/SE (10.77 vs. 7.08%, *p* < 0.001), major bleeding (11.26 vs. 7.51%, *p* < 0.001), and in-hospital any-cause death (5.84 vs. 3.94%, *p* = 0.01). The Kaplan-Meier curves (Figures 2A,C) also showed significant between-group differences in the cumulative incidence of stroke and major bleeding (log-rank *p* = 0.01 and *p* = 0.03, respectively). Among the 250 patients with eGFR <30 ml/min/1.73 m^2^ (Figures 2B,D), those treated with apixaban tended to have fewer events of stroke/SE and major bleeding (log-rank *p* = 0.09 and *p* = 0.06, respectively). Meanwhile, there was no significant between-group difference in the rate of in-hospital any-cause death.

**Table 2:** Study outcomes in the matched cohort and in the advanced CKD subgroup.

	**Apixaban-warfarin matched cohort (*****n*** **= 3,250)**				**Baseline eGFR < 30 (*****n*** **= 250)**			
	**Event**	**Warfarin**	**Apixaban**	***p*-value**	**Event**	**Warfarin**	**Apixaban**	***p*-value**
**Stroke/systemic embolism**, ***n*** **(%)**	290	175 (10.77)	115 (7.08)	0.0002	21	15 (11.45)	6 (5.04)	0.0681
Ischemic or uncertain stroke	222	111 (6.83)	111 (6.83)	1.0000	16	10 (7.63)	6 (5.04)	0.4031
Systemic embolism	77	72 (4.43)	5 (0.31)	<.0001	6	6 (4.58)	0 (0.00)	
**Major bleeding**, ***n*** **(%)**	305	183 (11.26)	122 (7.51)	0.0002	44	30 (22.90)	14 (11.76)	0.0209
Intracranial	66	36 (2.22)	30 (1.85)	0.4556	6	4 (3.05)	2 (1.68)	0.4788
Ocular	5	3 (0.18)	2 (0.12)	0.6545	0	0 (0.00)	0 (0.00)	
Intraabdominal	2	2 (0.12)	0 (0.00)		0	0 (0.00)	0 (0.00)	
Hematuria	20	12 (0.74)	8 (0.49)	0.3696	2	1 (0.76)	1 (0.84)	0.9456
Gastrointestinal	213	128 (7.88)	85 (5.23)	0.0023	36	25 (19.08)	11 (9.24)	0.0269
Other sites	8	7 (0.43)	1 (0.06)	0.0337	1	1 (0.76)	0 (0.00)	
**Other outcomes**, ***n*** **(%)**								
In-hospital death, *n* (%)	159	95 (5.85)	64 (3.94)	0.0117	24	13 (9.92)	11 (9.24)	0.8554
Pneumonia	302	175 (10.77)	127 (7.82)	0.0037	34	19 (14.50)	15 (12.61)	0.6618
Hip fracture	26	17 (1.05)	9 (0.55)	0.1152	4	3 (2.29)	1 (0.84)	0.3616

*eGFR, estimated glomerular filtration rate, ml/min/1.73 m^2^*.

### Stroke/Systemic Embolism

**Figure 3:**
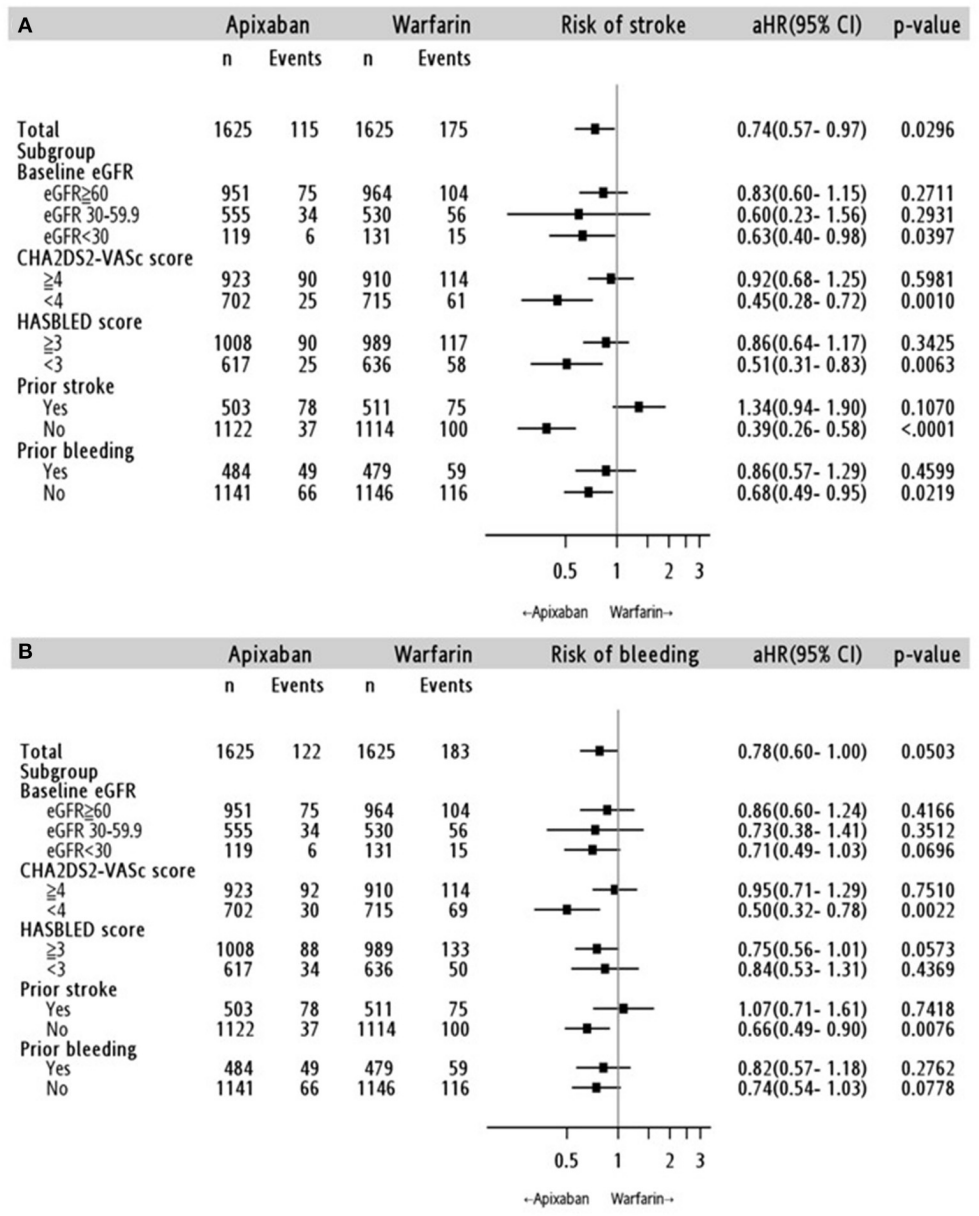
Stratified analyses for the risk of **(A)** ischemic stroke/systemic embolism and **(B)** major bleeding in the apixaban-warfarin matched cohort. aHR: adjusted hazard ratio; 95% CI, 95% confidence interval; eGFR: estimated glomerular filtration rate (ml/min/1.73 m^2^).

Cox proportional hazards regression analysis showed that apixaban treatment was associated with a lower risk of ischemic stroke/SE than warfarin treatment (adjusted hazard ratio [aHR]: 0.74; 95% confidence interval [CI]: 0.57–0.97; *p* = 0.03) (Figure 3A, Supplementary Table 2). The relative effect on ischemic stroke/SE prevention was not influenced by the apixaban dose (standard dose: aHR, 0.71; 95% CI, 0.50–1.01; *p* = 0.06; reduced dose: aHR, 0.77; 95% CI, 0.57–1.05; *p* = 0.09) (Table 3, Supplementary Table 2)

**Table 3:** Study outcomes by apixaban dose.

	Apixaban (reduced dose)^*^ vs. Warfarin			Apixaban (standard dose)^*^ vs. Warfarin		
	**aHR**	**95% CI**	***p-value***	**aHR**	**95% CI**	***p-value***
**Stroke/SE**						
Overall	0.77	(0.57, 1.05)	0.0955	0.71	(0.50, 1.01)	0.0575
**Baseline eGFR group**						
≧60	0.86	(0.57, 1.27)	0.3304	0.82	(0.54, 1.20)	0.3175
30–59.9	0.68	(0.42, 1.09)	0.2522	0.48	(0.20, 1.00)	0.0605
<30	0.66	(0.23, 1.65)	0.7231	-	-	-
**Major bleeding**						
Overall	0.84	(0.63, 1.12)	0.2286	0.66	(0.45, 0.96)	0.0287
**Baseline eGFR group**						
≧60	1.03	(0.67, 1.57)	0.5912	0.70	(0.43, 1.11)	0.0856
30–59.9	0.72	(0.47, 1.07)	0.1758	0.69	(0.36, 1.21)	0.2677
<30	0.81	(0.41, 1.53)	0.3807	-	-	-

* *Apixaban standard dose: 10 mg/day, reduced dose: 2.5–5 mg/day; -: aHR was not available because no event was observed in the advanced CKD subgroup (eGFR <30 ml/min/1.73 m^2^). aHR: adjusted hazard ratio; 95% CI, 95% confidence interval; eGFR: estimated glomerular filtration rate (ml/min/1.73 m^2^)*.

### Major Bleeding

In the entire cohort, gastrointestinal bleeding was high in both the apixaban and warfarin groups (5.23 vs. 7.88%), following by intracranial bleeding (1.85 vs. 2.22%) and hematuria (0.49 vs. 0.74%) in Table 2. Apixaban reduced the risk of major bleeding by 22%, but the difference did not reach statistical significance (aHR, 0.78; 95% CI, 0.60–1.00; *p* = 0.05). The standard dose of apixaban significantly lowered the risk of major bleeding (aHR, 0.66; 95% CI, 0.45–0.96; *p* = 0.03) than warfarin, but the reduced dose of apixaban didn't exhibit significantly difference in major bleeding (aHR, 0.84; 95% CI, 0.63–1.12; *p* = 0.23) (Table 3, Supplementary Table 3).

### Kidney Function

The results according to the eGFR classification were consistent with the main analysis (Figures 3A,B). In the advanced CKD subgroup, apixaban initiation was significantly associated with a lower risk of stroke/SE (aHR: 0.63; 95% CI: 0.40–0.98, *p* = 0.04), but not for major bleeding (aHR: 0.71; 95% CI: 0.49–1.03; *p* = 0.70). Meanwhile, there was no significant difference in the risk of stroke/SE or major bleeding outcomes between apixaban and warfarin in the mild and moderate CKD subgroups. Further stratified analyses to investigate the impact of apixaban dose on the association between kidney function and risk of major bleeding showed inconclusive findings because there was no event in the advanced CKD subgroup (Table 3).

### Other Subgroup and Sensitivity Analyses

The relative effects of apixaban according to a history of stroke and CHA_2_DS_2_-VASc score at baseline (<4 and ≥4), prior major bleeding, and HAS-BLED score at baseline (<3 and ≥3) are shown in Figures 3A,B. In general, apixaban was associated with more favorable outcomes than warfarin in patients without a history of stoke, CHA2DS2-VASc score <4, without history of major bleeding, and with a HAS-BLED score <3. In addition, the choice of OAC did not increase the risk of pneumonia (aHR: 0.99; 95% CI: 0.76–1.28; *p* = 0.91) and hip fracture (aHR: 0.71; 95% CI: 0.29-1.76; *p* = 0.44) (Supplementary Table 4).

## Discussion

Real-world evidence on the benefit of apixaban in AF patients with CKD without dialysis is limited. This study found that apixaban lowers the risk of ischemic stroke or SE by 26% in AF patients with CKD and by 37% in those with advanced CKD. Meanwhile, although the rate of major bleeding was lower in the apixaban group than it was in the warfarin group, the difference was not statistically significant in the overall cohort and across the eGFR groups. Subgroup analysis according to apixaban doses showed that a standard dose of 10 mg/day was associated with a 34% lower risk of major bleeding.

The first real-word study on apixaban vs. warfarin use in CKD patients was published in 2017. The study, which included 146 patients with CrCl <25 mL/min or serum creatinine >2.5 mg/dL, found no significant differences with respect to major bleeding or thromboembolic events between apixaban and warfarin treatments (29). A recent subgroup analysis from the Apixaban for Reduction in Stroke and Other Thromboembolic Events in Atrial Fibrillation (ARISTOTLE) trial also showed no significant difference in stoke or SE prevention and all-cause mortality between apixaban and warfarin in patients with CrCl 25–30 mL/min (30). A US Medicare population cohort of 22,739 AF patients with group 3, 4, and 5 CKD compared apixaban, rivaroxaban, and dabigatran with warfarin in CKD patients and found that apixaban was associated with the lowest risk of stroke/SE (31). However, most of the patients (>80%) had group 3 CKD (eGFR between 30 and 59), and the patients were identified using diagnostic codes, limiting the generalizability of the findings to the advanced CKD patients. In the present study, the incidence rate of ischemic stroke/SE in patients with advanced CKD is comparable to that in previous observational studies (18, 29). The relatively large sample size of CKD patients in the present study and the data from a national representative database provide robust real-world evidence on the relative effect of apixaban in comparison to that of warfarin on stroke/SE prevention in a heterogeneous CKD population.

The apixaban dose is an important influencing factor of its efficacy and safety in patients with CKD. Although the apixaban label indicates a dose of 5 mg twice daily for non-valvular AF, patients are recommended to take apixaban 2.5 mg twice daily if they meet at least two of the following characteristics: age ≥ 80 years, body weight ≤ 60 kg, and serum creatinine ≥1.5 mg/dL (32). In the secondary analyses of the ARISTOTLE trial, the risk of stroke/SE was 23% lower in standard dose apixaban (5 mg twice daily) than in warfarin, whereas there was no significant difference between reduced dose apixaban and warfarin (33). With respect to major bleeding, the risk was lower in apixaban than in warfarin irrespective of the apixaban dose, with the benefit being more profound in patients who were older, weighed less, and had serum creatinine ≥1.5 mg/dL (or lower CrCl) (30). Apixaban was also reported to be associated with lower rates of major bleeding than warfarin among patients with CrCl of 25–30 ml/min (30). Overall, data from the ARISTOTLE trial support that standard dose apixaban (5 mg twice daily) has a better pharmacokinetic distribution in patients with CrCl 25–30 ml/min than in those with higher CrCl (>30 ml/min) (30). The findings collectively suggest that the standard dose of apixaban may be safe in patients with CKD.

Given the low rate of OAC use in patients with eGFR <15 ml/min/1.73 m^2^ in the current study, we were unable to evaluate the relative benefits and disadvantages of apixaban in comparison to those of warfarin. However, the results support that the risk of stroke/SE was lower in apixaban treatment than in warfarin treatment in patients with eGFR <30 ml/min/1.73 m^2^, and apixaban was more beneficial in patients with low eGFR values than in those with high eGFR values, consistent with previous findings (30, 34). However, the effect of apixaban dose on the association between kidney function and risk of stroke/SE and major bleeding was not clarified in the present study.

The ARISTOLE trial suggested that apixaban was not inferior to warfarin as it had a mean time in therapeutic range (TTR) of 62% and an international normalized ratio (INR) of 2.0–3.0 (4). A subanalysis of the ARISTOTLE trial showed relatively lower mean TTR in East Asians (mean 27.2 ± 11.07) compared to those of non-East Asians (30.1 ± 14.29), and the duration with an international normalized ratio (INR) of <2 was longer in East Asians (28.6%) than in non-East Asians (18%) (35). Furthermore, the level of TTR varies between different countries (44–77%), and according to a dabigatran multinational trial, the mean TTR was lowest (44%) in Taiwan (36). In the present study, the mean INR was 1.97 (±1.01) during the total follow up period among patients with at least one INR values in the warfarin group (*n* = 1,511), and these patients had a higher rate of intracranial hemorrhage compared to those of patients in the apixaban group. The high rate of intracranial hemorrhage in patients with a lower INR compared to those of controls is similar to the findings in Asian patients in the apixaban (35) and dabigatran multinational trials (36).

The effectiveness and safety of using warfarin is associated with its optimum therapeutic INR control. We noted a high rate of systematic embolism in patients treated with warfarin with great INR fluctuations from the mean value of 3.14 (±1.67) to 1.72 (±0.72) over the follow-up period (Supplementary Figure 1). The high variability of INR may be because of poor adherence or difficult management in some warfarin users (35, 37). Low intensity of anticoagulation is a common practice in Taiwan. Regarding the interpretation of these study results, it is important to address the differences in the relative effect of DOACs vs. warfarin between Asian and non-Asian populations (35, 36).

Of note, gastrointestinal bleeding and intracranial hemorrhage were the most common major bleedings in this study cohort, and this is consistent with the reports of a population-based observational study in Taiwan (1.81 per 100 person-years for gastrointestinal bleeding, 1.53 per 100 person-years for intracranial hemorrhage) (38). The population-based observational study and the meta-analyses of multinational randomized trials suggested that all DOACs can reduce overall major bleeding risk, but only apixaban was superior to warfarin in terms of fewer rates of major bleeding (39) or gastrointestinal bleeding (38). The reason for the differences in the risk of gastrointestinal bleeding between DOACs requires further research (40). In the present study, only patients with standard-dose apixaban (vs. warfarin) revealed statistically significant reduction in overall major bleeding. Further research into precise apixaban dosing could support the use of apixaban as an alternative to warfarin in patients with chronic kidney disease and atrial fibrillation.

The present study has limitations. First, like other retrospective studies, biases due to residual confounding may not have been eliminated. The present study applied hospitalization for pneumonia and hip fracture as negative control outcomes to ensure the robustness of the relative effects of apixaban in comparison to those of warfarin. There were no associations between both negative control outcomes and treatment choices, which indicated that there was no evidence for unmeasured confounding bias. Second, there was a high proportion of patients who received reduced-dose apixaban. This could be because the patients were older, had worse kidney function, and a higher HAS-BLED score (Supplementary Table 5), as is characteristics of the Asian population (41, 42). Reduced-dose DOACs is common in real-world practice, especially in Asians (43–45). Third, the results may be applicable only in Taiwanese or Asian populations and have limited generalizability to the overall population of CKD patients. However, the clinical practice pattern in the study setting is likely to follow international clinical guidelines and could help improve understanding of the benefit/disadvantage of anticoagulation in patients with kidney dysfunction. Further, we measured kidney function using the Taiwan version of the MDRD formula (28), as is routine practice in Taiwan. MDRD-based eGFR values could be not the same as CrCl in ARISTOTLE trial (46). The current study findings may help establish the appropriate apixaban dose in high-risk patients, such as those with advanced CKD and the elderly, according to kidney function estimated with the MDRD formula.

In conclusion, the risk of stroke/SE is lower in AF patients receiving apixaban treatment than in those receiving warfarin treatment, and the benefits of apixaban are also noted in patients with advanced CKD (eGFR <30 ml/min/1.73 m^2^). Further, compared to warfarin, both standard and reduced dose of apixaban do not increase the risk of major bleeding. Our findings highlight the importance of appropriate anticoagulation treatment in patients with AF and kidney disease.

## Data Availability Statement

The raw data supporting the conclusions of this article will be made available by the authors, without undue reservation.

## Ethics Statement

The studies involving human participants were reviewed and approved by Institutional Review Board of Chang Gung Medical Foundation at Taipei, Taiwan. Written informed consent for participation was not required for this study in accordance with the national legislation and the institutional requirements.

## Author Contributions

C-MF, L-CL, and C-NH: conceptualization and wrote manuscript—original draft preparation. C-MF and C-NH: formal analysis, methodology, funding acquisition. All authors: investigation, validation, visualization, wrote manuscript—review, and editing.

## Funding

This work was supported by the Ministry of Science and Technology, Taiwan, under Grants MOST 107-2622-E-182A-001-CC3 and 109-2622-E-182A-001-CC3 (recipient: C-NH), and Kaohsiung Chang Gung Memorial Hospital, Taiwan under Grant CFRPG8J0041 (recipient: C-MF) and CMRPG8E0463 (recipient: C-NH).

## Conflict of Interest

The authors declare that the research was conducted in the absence of any commercial or financial relationships that could be construed as a potential conflict of interest.

## Publisher's Note

All claims expressed in this article are solely those of the authors and do not necessarily represent those of their affiliated organizations, or those of the publisher, the editors and the reviewers. Any product that may be evaluated in this article, or claim that may be made by its manufacturer, is not guaranteed or endorsed by the publisher.
