# Supplementary material for: Apixaban vs. Warfarin in Atrial Fibrillation Patients With Chronic Kidney Disease
Source: Front Cardiovasc Med. 2021 Oct 18;8:752468. doi: 10.3389/fcvm.2021.752468 (PMC8558356; doi:10.3389/fcvm.2021.752468)
Supplement: Supplementary file 1 [file Data_Sheet_1.docx]

**Apixaban versus Warfarin in Atrial Fibrillation Patients with Chronic Kidney Disease**

**Supplemental materials**

Supplemental Table 1. Codes for clinical condition and medication used in this study

Supplemental Table 2. Cox proportional hazards model for ischemic stroke or systemic embolism

Supplemental Table 3. Cox proportional hazards model for major bleeding

Supplemental Table 4. Cox proportional hazards model for pneumonia and hip fracture

Supplemental Table 5. Characteristics of apixaban users with standard or reduced initial dose

Supplemental Figure 1. The mean international normalized ratios in patients treated with warfarin who developed systemic embolism (n=70)

Supplemental Table 1. Codes for clinical condition and medication used in this study

| **Condition** | **Coding system /Code** |
| --- | --- |
| Atrial fibrillation/atrial flutter | ≥ 2 visits in outpatient/emergency department within 365 days and apart ≥ 28 days or ≥ 1 hospitalization for the disease defined by International Classification of Diseases (ICD)-9/10 codes:  ICD-9-CM: 427.31, 427.32; ICD10-CM: I48 |
| Oral anticoagulant treatment | New user was identified by Anatomical Therapeutic Chemical Classification system (ATC) code for warfarin or apixaban initiation:  warfarin: B01AA03; dabigatran: B01AE07; rivaroxaban:B01AF01; apixaban B01AF02; edoxaban:B01AF03 |
| **Outcome** | **≥1 hospitalizations for the disease after the index date** |
| **Stroke/systemic embolism (SE)** |  |
| Ischemic or uncertain stroke | ICD-9-CM: 433.x-437.x ICD10-CM: I63, I65, I66, I67.2, I67.5-I67.82, I67.841-I67.848, I67.89-I67.9, I68, G45.0-G45.2, G45.4-G45.9, G46, Z86.73 |
| SE, pulmonary embolism | ICD-9-CM: 453.x, 415.x ICD10-CM: I82.2-I82.9, I82.A, I82.B, I82.C, Z86.71, I26.02, I26.09, I26,9, I27.82 |
| **Major bleeding [1-5]** |  |
| Intracranial | ICD-9-CM: 430, 431, 432.0, 432.1, 432.9, 852.0, 852.2, 852.4, 853.0 ICD10-CM: I60, I61, I62, S06.340A-S06.349A, S06.350A-S06.359A, S06.360A-06.369A, S06.4X0A-S06.4X9A, S06.5X0A-S06.5X9A, S06.6X0A-S06.6X9A |
| Ocular | ICD-9-CM: 360.43, 362.81, 363.6, 364.4, 377.42, 379.23, 376.32, 372.72 ICD10-CM: H44.8, H35.6, H31.3, H21.0, H47.02, H43.1, H05.23, H11.3 |
| Gastrointestinal | ICD-9-CM: 530.7, 531, 531.2, 531.4, 531.6, 532, 532.2, 532.4, 532.6, 533, 533.2, 533.4, 533.6, 534, 534.2, 534.4, 534.6, 569.3, 535.01, 535.11, 535.21, 535.31, 535.41, 535.51, 535.61, 535.71, 537.83, 537.84, 562.02, 562.03, 562.12, 562.13, 569.85, 578 ICD10-CM: K22.6, K25-K28, K29.01, K29.21, K29.31, K29.41, K29.51, K29.61, K29.71, K29.81, K29.91, K31.811, K31.82, K52.81, K55.21, K56.60, K56.60, K57.01, K57.11, K57.13, K57.21, K57.31, K57.33, K57.81, K57.91, K57.93, K62.5, K92.0, K92.1, K92.2 |
| Intra-abdominal | ICD-9-CM: 568.81, 866.01, 866.02, 866.11, 866.12 ICD10-CM: K66.1, S37.011A, S31.001A |
| Hematuria | ICD-9-CM: 599.7 ICD10-CM: R31, N30.01, N30.21, N30.31, N30.81, N30.91 |
| Other sites | ICD-9-CM: 336.1(spinal hemorrhage), 363.6, 372.72, 376.32, 377.42, 379.23 (Ocular bleeding), 593.81(renal artery hemorrhage), 866.01, 866.11(kidney hematoma), 866.02, 866.12(kidney laceration), 719.1, 729.92 (intra-articular hemorrhage), 423.0(pericardial hemorrhage), 772.5(Adrenal gland bleeding)  ICD10-CM: G95.11, G95.19(spinal hemorrhage), H05.23, H11.3, H31.3, H43.1, H47.02(Ocular bleeding), I31.2 (pericardial hemorrhage), M25.0(intra-articular hemorrhage), N28.0 (kidney hematoma), S31.001A, S37.011A, S37.012A, S37.019A, S37.021A, S37.022A, S37.029A, S37.031A, S37.032A, S37.039A, S37.041A, S37.042A, S37.049A, S37.051A, S37.052A, S37.059A (Injury of kidney), P54.4 (Adrenal bleeding). |
| Pneumonia | ICD-9-CM: 480-488/ ICD10-CM: J09X1, J10.0, J11.0, J12-J16, J18 |
| Hip fracture | ICD-9-CM: 820-821/ ICD10-CM: S72 |
| **Baseline comorbidity** | **≥2 visits in the outpatient/emergency department within 365 days prior to the index date and ≥28 days apart or ≥1 hospitalization for the disease** |
| Charlson Comorbidity Index | 1.Acute myocardial infarction =ICD-9:410,412/ICD-10:I21,I22, I252; 2.Congestive heart failure=ICD-9:428/ICD-10:I50; 3.Peripheral vascular disease=ICD-9: 441, 443.9, 785.4, V434/ICD-10:I71,I790, R02, Z958, Z959; 4.Cerebral vascular accident=ICD-9:430-438/ICD-10: I60, I61, I62, I63, I65, I66,G450, G451, G452, G458, G459, G46, I64, G454, I670, I671, I672, I674, I675, I676, I677 I678, I679, I681, I682, I688, I69;  5.Dementia=ICD-9:290/ICD-10: F00, F01, F02, F051; 6.Pulmonary disease=ICD-9: 490, 491, 492, 493, 494, 495, 496, 500, 501, 502, 503, 504, 505/ICD-10: J40, J41, J42, J44, J43, J45, J46, J47, J67, J44, J60, J61, J62, J63, J66, J64, J65; 7.Connective tissue disorder=ICD-9: 7100, 7101, 7104, 7140, 7141, 7142, 71481/ 5171, 725/ICD-10: M32, M34, M332, M053, M058, M059, M060, M063, M069, M050, M052, M051, M353;  8.Peptic ulcer=ICD-9: 531, 532, 533, 534/ICD-10: K25, K26, K27, K28; 9.Liver disease=ICD-9: 5712, 5714, 5715, 5716/ICD-10: K702, K703, K73, K717, K740, K742, K746, K743, K744, K745;  10. Diabetes=ICD-9: 2500, 2501, 2502, 2503, 2507/ICD-10: E109, E119, E139, E149, E101, E111, E131, E141, E105, E115, E135, E145; 11. Diabetes complications=ICD-9: 2504, 2505, 2506/ICD-10: E102, E112, E132, E142 E103, E113, E133, E143 E104, E114, E134, E144;  12. Paraplegia=ICD9:342, 3441/ICD-10: G81 G041, G820, G821, G822; 13.Renal disease=ICD9: 582, 5830, 5831, 5832, 5833, 5835, 5836, 5837, 5834, 585, 586,588/ICD-10: N03, N052, N053, N054, N055, N056, N072, N073, N074, N01, N18, N19, N25;  14. Cancer=ICD-9: 14-16, 18, 170-172, 174-176, 179, 190-194, 195.0-195.55, 195.8, 200-208/ICD-10: C0-C3, C40,C41, C43, C45-C49, C5, C6, C70-C76, C80-C85, C883, C887, C889, C900, C901, C91, C92, C93, C940-C943, C9451, C947, C95, C96; 15. Metastatic cancer=ICD-9: 196-198, 1990, 1991/ICD-10: C77-C80;  16. Severe liver disease=ICD-9: 5722, 5723, 5724, 5728/ICD-10:K729, K766, K767, K721; 17. HIV=ICD-9:042,043,044/ICD-10: B20, B21, B22, B23, B24. |
| CHA2DS2-VASc score | score=[(Congestive heart failure+ Hypertension+Age 65-74years+Dabetes mellitus+Vascular disease+Sex_female)x1]+[(Age ≥75+Stroke)*2]  C=ICD-9:428 or ICD10: I11.0, I50, I97.1; D=ICD-9:250 or ICD-10: E10, E11, E12, E13, E14;V=ICD-9:443 or ICD-10: I21, I22, I25.2, I70.0, I70.1, I70.2, I70.8, I70; S/TIA/TE=ICD-9: 433, 434, 435, 453, 415 or ICD-10:I63, I64,G45. |
| HASBLED score | score=[Hypertension+abnormal renal/liver function(0, 1 or 2)+stroke+ bleeding history or predisposition+labile INR (international normalized ratio>3)+age >65 years+concomitant use of NSAIDs or alcohol (0,1 or 2); whenever data for the disease, medication use and INR were available]  Renal=ICD-9: ICD9: 403, 404, 580 -589; or ICD-10:N280; Liver=ICD-9:570-573 or ICD-10: B15-B19, C22, C64-C65, D684C, E102, E112, E132, E142, I12-I13, I15, I982B, K70-K77, N1, N25-N26, N289, Q61, Z940, Z944, Z992; Stroke (inpatient)=ICD-9:433, 434 or ICD-10: G458, G459, I63, I64, I74; Bleeding (inpatient)= ICD9: 531.0x, 531.2x, 531.4x, 531.6x, 532.0x, 532.2x, 532.4x, 532.6x, 533.0x, 533.2x, 533.4x, 533.6x, 534.0x, 534.2x, 534.4x, 534.6x, 535.01, 535.11, 535.21, 535.31, 535.41, 535.51, 535.61, 537.83, 456.0, 456.20, 530.7, 530.82, 578.0, 455.2, 455.5, 455.8, 562.02, 562.03, 562.12, 562.13, 568.81, 569.3, 569.85, 578.1, 578.9, 593.81, 599.7, 623.8, 626.2, 626.6, 430, 431, 432, 432.0, 432.1, 432.9, 852.0, 852.2, 852.4, 853.0, 423.0, 459.0, 568.81, 719.1x, 784.7, 784.8, 786.3 or critical site bleeding (ICD-9: 430, 431, 432, 852.0, 852.2, 852.4, 853.0, 336.1, 363.6, 372.72, 376.32, 377.42, 379.23, 719.1, 729.92, 729.97, 423.0, 593.81, 772.5, 866.01, 866.02, 866.11, 866.12) or ICD-10: I60-I62, I690-I692, J942, K250, K254, K260, K264, K270, K280, K920-K922, N02, R04, R31, S064-S066; Use of NSAID or anti-platelet: M01A or ATCB01AC; Alcohol=ICD-9: 291.0-291.9; 303.x; 305.0; 357.5; 425.5;571.0-571.3 or ICD-10: F1, K70, E52, T51, K860, E244, G312, I426, O354, Z714, Z721, G312, G621, G721, K292, L278A. |
| Hypertension in CHA2DS2-VASc and HASBLED score | (1) medication use duration>=90 days in outpatient setting;  ATC: C02A-C02D, C02L, C03A, C03B, C03D, C03E, C03X, C04, C05, C07-C09.  (2) or ICD-9: 401-405; ICD-10: I10, I11, I12, I13, I15. |
| **Prior medication use** | **≥28 days of supply within 365 days prior to the index date for the medication use defined by ATC code** |
| Lipid-lowering agent | C10AA,C10BA, C10AB, C10AC, C10AD,C10AX, C10BX |
| Antidiabetic agent | A10A, A10B |
| Anti-hypertension | C09A, C09B, C09C, C09D, C09X, C03, C07, C08 |
| Anti-platelet agent | B01AC06, B01AC04, B01AC24, B01AC07, B01AC22, B01AC30, B01AC91 |
| Amiodarone | C01BD01, C01BD07 |
| Digoxin | C01AA05 |
| Non-steroidal anti-inflammatory drugs | M01AA, M01AX, M01AB, M01AC, M01AE, M01AG, M01AH |
| Gastric antacids | A02BA, A02BC |

The major bleeding was defined as hospitalization with any diagnosis of ICD-9/10-CM codes for bleeding events in order to comparable with the International Society of Thrombosis and Haemostasis major bleeding definitions.

References

1. Chang SH et al., Association between use of non–vitamin K oral anticoagulants with and without concurrent medications and risk of major bleeding in nonvalvular atrial fibrillation. Jama 2017; 318 (13):1250-1259.

2. Schulman S and Kearon C. Definition of major bleeding in clinical investigations of antihemostatic medicinal products in non-surgical patients. J Thromb Haemost, 2005. 3(4): 692-4.

3. Yu HT., et al., Clinical Significance of Hematuria in Atrial Fibrillation With Oral Anticoagulation Therapy. Circ J, 2017. 81(2):158-164.

4. Limdi NA et al. Kidney function influences warfarin responsiveness and hemorrhagic complications. J Am Soc Nephrol, 2009. 20(4): 912-21.

Supplemental Table 2. Cox proportional hazards regression model for ischemic stroke or systemic embolism

|  |  | **No of patient** | **No of event** | **Main analysis** | | | | |  | **Apixaban subgroup analysis** | | | | |
| --- | --- | --- | --- | --- | --- | --- | --- | --- | --- | --- | --- | --- | --- | --- |
| **Variable** | |  |  | **aHR** | **95% CI** | | | *p-value* |  | **aHR** | **95% CI** | | | *p-value* |
| **Treatment** | |  |  |  |  |  |  |  |  |  |  |  |  |  |
|  | Warfarin | 1625 | 175 | 1.00 |  | | |  |  | - | - |  | - | - |
|  | Apixaban | 1625 | 115 | 0.74 | (0.57 |  | 0.97) | 0.0296 |  | - | - |  | - | - |
| **Treatment** | |  |  |  |  |  |  |  |  |  |  |  |  |  |
|  | Warfarin | 1625 | 175 | - | - |  | - | - |  | 1.00 |  | | |  |
|  | **Apixaban (reduced dose 2.5-5 mg/day)** | **915** | **69** | **-** | **-** |  | **-** | **-** |  | **0.77** | **(0.57** |  | **1.05)** | **0.0955** |
|  | **Apixaban (standard dose 10 mg/day)** | **710** | **46** | **-** | **-** |  | **-** | **-** |  | **0.71** | **(0.50** |  | **1.01)** | **0.0575** |
| **Age, years** | |  |  |  |  |  |  |  |  |  |  |  |  |  |
|  | <40 | 23 | 1 | 1.00 |  | | |  |  | 1.00 |  | | |  |
|  | 40-64 | 502 | 36 | 1.50 | (0.21 |  | 11.03) | 0.6887 |  | 1.50 | (0.20 |  | 10.98) | 0.6915 |
|  | 65-74 | 1007 | 73 | 1.47 | (0.20 |  | 10.69) | 0.7027 |  | 1.46 | (0.20 |  | 10.61) | 0.7081 |
|  | ≥75 | 1718 | 180 | 2.15 | (0.30 |  | 15.52) | 0.4476 |  | 2.12 | (0.29 |  | 15.31) | 0.4570 |
| **Sex** | |  |  |  |  |  |  |  |  |  |  |  |  |  |
|  | Male | 1864 | 156 | 0.88 | (0.69 |  | 1.11) | 0.2766 |  | 0.88 | (0.69 |  | 1.12) | 0.2811 |
|  | Female | 1386 | 134 | 1.00 |  | | |  |  | 1.00 |  | | |  |
| **Baseline estimated glomerular filtration rate (eGFR), ml/min/1.73 m^2^** | | |  |  |  |  |  |  |  |  |  |  |  |  |
|  | ≧60 | 1915 | 179 | 1.00 |  | | |  |  | 1.00 |  | | |  |
|  | 30-59.9 | 1085 | 90 | 0.75 | (0.56 |  | 1.01) | 0.0555 |  | 0.75 | (0.56 |  | 1.00) | 0.0518 |
|  | <30 | 250 | 21 | 0.63 | (0.37 |  | 1.08) | 0.0943 |  | 0.63 | (0.37 |  | 1.07) | 0.0891 |
| **Baseline international normalized ratio** | |  |  |  |  |  |  |  |  |  |  |  |  |  |
|  | <1.5 | 2080 | 181 | 1.00 |  | | |  |  | 1.00 |  | | |  |
|  | 1.5-2 | 324 | 31 | 0.86 | (0.58 |  | 1.29) | 0.4721 |  | 0.86 | (0.58 |  | 1.29) | 0.4706 |
|  | >2 | 390 | 49 | 1.08 | (0.76 |  | 1.53) | 0.6672 |  | 1.08 | (0.76 |  | 1.53) | 0.6690 |
|  | Missing | 456 | 29 | 0.96 | (0.64 |  | 1.44) | 0.8526 |  | 0.97 | (0.65 |  | 1.45) | 0.8653 |
| **Baseline comorbid condition** | |  |  |  |  |  |  |  |  |  |  |  |  |  |
|  | Ischemic stroke/systemic embolism | 1014 | 153 | 2.40 | (1.87 |  | 3.08) | <.0001 |  | 2.40 | (1.87 |  | 3.09) | <.0001 |
|  | Major bleeding | 963 | 108 | 1.25 | (0.96 |  | 1.61) | 0.0972 |  | 1.25 | (0.96 |  | 1.62) | 0.0936 |
|  | Hypertension | 2363 | 212 | 0.82 | (0.62 |  | 1.09) | 0.1619 |  | 0.82 | (0.62 |  | 1.08) | 0.1594 |
|  | Renal disease | 627 | 70 | 1.79 | (1.28 |  | 2.51) | 0.0007 |  | 1.79 | (1.28 |  | 2.51) | 0.0007 |
|  | Liver diseases/Severe liver diseases | 236 | 22 | 0.97 | (0.62 |  | 1.51) | 0.8969 |  | 0.97 | (0.62 |  | 1.51) | 0.8857 |
|  | Diabetes mellitus | 1030 | 95 | 1.18 | (0.83 |  | 1.66) | 0.3573 |  | 1.18 | (0.83 |  | 1.66) | 0.3528 |
|  | Heart failure | 1064 | 85 | 1.03 | (0.79 |  | 1.34) | 0.8330 |  | 1.03 | (0.79 |  | 1.34) | 0.8346 |
|  | Peripheral vascular disease | 111 | 14 | 1.45 | (0.83 |  | 2.53) | 0.1918 |  | 1.44 | (0.82 |  | 2.51) | 0.2018 |
| **Prior medications uses** | |  |  |  |  |  |  |  |  |  |  |  |  |  |
|  | Lipid-lowering agent | 817 | 66 | 0.80 | (0.60 |  | 1.08) | 0.1421 |  | 0.80 | (0.60 |  | 1.08) | 0.1437 |
|  | Glucose-lowering agent | 682 | 59 | 0.83 | (0.55 |  | 1.25) | 0.3715 |  | 0.83 | (0.55 |  | 1.25) | 0.3715 |
|  | Anti-hypertension | 2508 | 217 | 0.97 | (0.71 |  | 1.32) | 0.8529 |  | 0.97 | (0.71 |  | 1.32) | 0.8498 |
|  | Anti-platelet agent | 1398 | 120 | 0.81 | (0.62 |  | 1.05) | 0.1084 |  | 0.81 | (0.62 |  | 1.05) | 0.1071 |
|  | Amiodarone | 603 | 47 | 0.92 | (0.67 |  | 1.27) | 0.6107 |  | 0.92 | (0.66 |  | 1.27) | 0.5997 |
|  | Digoxin | 295 | 14 | 0.52 | (0.30 |  | 0.90) | 0.0196 |  | 0.52 | (0.30 |  | 0.90) | 0.0193 |
|  | Non-steroidal anti-inflammatory drugs | 374 | 47 | 1.35 | (0.98 |  | 1.87) | 0.0652 |  | 1.35 | (0.98 |  | 1.87) | 0.0652 |
|  | Gastric antacids | 844 | 87 | 1.21 | (0.92 |  | 1.59) | 0.1744 |  | 1.20 | (0.92 |  | 1.58) | 0.1822 |

aHR=adjusted hazard ratio, CI=confidence interval

Supplemental Table 3. Cox proportional hazards regression model for major bleeding

|  |  | **No of  patient** | **No of  event** | **Main analysis** | | | | |  | **Apixaban subgroup analysis** | | | | |
| --- | --- | --- | --- | --- | --- | --- | --- | --- | --- | --- | --- | --- | --- | --- |
| **Variable** | |  |  | **aHR** | **95% CI** | | | *p-value* |  | **aHR** | **95% CI** | | | *p-value* |
| **Treatment** | |  |  |  |  |  |  |  |  |  |  |  |  |  |
|  | Warfarin | 1625 | 183 | 1.00 |  | | |  |  | - | - |  | - | - |
|  | Apixaban | 1625 | 122 | 0.78 | (0.60 |  | 1.00) | 0.0503 |  | - | - |  | - | - |
| **Treatment** | |  |  |  |  |  |  |  |  |  |  |  |  |  |
|  | Warfarin | 1625 | 183 | - | - |  | - | - |  | 1.00 |  | | |  |
|  | Apixaban (reduced dose 2.5-5 mg/day) | 915 | 85 | - | - |  | - | - |  | 0.84 | (0.63 |  | 1.12) | 0.2286 |
|  | Apixaban (standard dose 10 mg/day) | 710 | 37 | - | - |  | - | - |  | 0.66 | (0.45 |  | 0.96) | 0.0287 |
| **Age, years** | |  |  |  |  |  |  |  |  |  |  |  |  |  |
|  | <40 | 23 | 1 | 1.00 |  | | |  |  | 1.00 |  | | |  |
|  | 40-64 | 502 | 22 | 1.03 | (0.14 |  | 7.65) | 0.9796 |  | 1.02 | (0.14 |  | 7.58) | 0.9869 |
|  | 65-74 | 1007 | 82 | 1.80 | (0.25 |  | 13.02) | 0.5616 |  | 1.76 | (0.24 |  | 12.77) | 0.5751 |
|  | ≥75 | 1718 | 200 | 2.40 | (0.33 |  | 17.28) | 0.3857 |  | 2.30 | (0.32 |  | 16.57) | 0.4097 |
| **Sex** | |  |  |  |  |  |  |  |  |  |  |  |  |  |
|  | Male | 1864 | 169 | 1.08 | (0.86 |  | 1.37) | 0.5030 |  | 1.09 | (0.86 |  | 1.37) | 0.4924 |
|  | Female | 1386 | 136 | 1.00 |  | | |  |  | 1.00 |  | | |  |
| **Baseline estimated glomerular filtration rate, ml/min/1.73 m^2^** | | |  |  |  |  |  |  |  |  |  |  |  |  |
|  | ≧60 | 1915 | 136 |  |  | | |  |  |  |  | | |  |
|  | 30-59.9 | 1085 | 125 | 1.40 | (1.07 |  | 1.83) | 0.0139 |  | 1.38 | (1.05 |  | 1.80) | 0.0195 |
|  | <30 | 250 | 44 | 2.21 | (1.44 |  | 3.38) | 0.0003 |  | 2.16 | (1.41 |  | 3.31) | 0.0004 |
| **Baseline international normalized ratio** | |  |  |  |  |  |  |  |  |  |  |  |  |  |
|  | <1.5 | 2080 | 203 | 1.00 |  | | |  |  | 1.00 |  | | |  |
|  | 1.5-2 | 324 | 29 | 0.79 | (0.52 |  | 1.18) | 0.2494 |  | 0.79 | (0.52 |  | 1.18) | 0.2475 |
|  | >2 | 390 | 47 | 1.00 | (0.71 |  | 1.41) | 0.9887 |  | 0.99 | (0.70 |  | 1.41) | 0.9723 |
|  | Missing | 456 | 26 | 0.72 | (0.47 |  | 1.09) | 0.1154 |  | 0.72 | (0.48 |  | 1.10) | 0.1279 |
| **Baseline comorbid condition** | | |  |  |  |  |  |  |  |  |  |  |  |  |
|  | Ischemic stroke/systemic embolism | 1014 | 106 | 1.06 | (0.83 |  | 1.37) | 0.6437 |  | 1.07 | (0.83 |  | 1.37) | 0.6244 |
|  | Major bleeding | 963 | 138 | 1.77 | (1.37 |  | 2.27) | <.0001 |  | 1.78 | (1.38 |  | 2.29) | <.0001 |
|  | Hypertension | 2363 | 239 | 1.13 | (0.84 |  | 1.52) | 0.4077 |  | 1.13 | (0.84 |  | 1.51) | 0.4187 |
|  | Renal disease | 627 | 81 | 1.08 | (0.78 |  | 1.48) | 0.6581 |  | 1.07 | (0.78 |  | 1.47) | 0.6753 |
|  | Liver diseases/severe liver diseases | 236 | 28 | 1.16 | (0.78 |  | 1.73) | 0.4564 |  | 1.15 | (0.77 |  | 1.72) | 0.4899 |
|  | Diabetes mellitus | 1030 | 115 | 1.18 | (0.85 |  | 1.65) | 0.3314 |  | 1.18 | (0.85 |  | 1.65) | 0.3215 |
|  | Heart failure | 1064 | 116 | 1.27 | (0.99 |  | 1.62) | 0.0578 |  | 1.27 | (0.99 |  | 1.62) | 0.0605 |
|  | Peripheral vascular disease | 111 | 14 | 1.23 | (0.71 |  | 2.13) | 0.4607 |  | 1.22 | (0.70 |  | 2.11) | 0.4807 |
| **Prior medication use** | |  |  |  |  |  |  |  |  |  |  |  |  |  |
|  | Lipid-lowering agent | 817 | 76 | 0.94 | (0.71 |  | 1.25) | 0.6782 |  | 0.94 | (0.71 |  | 1.25) | 0.6874 |
|  | Glucose-lowering agent | 682 | 77 | 1.00 | (0.68 |  | 1.47) | 0.9966 |  | 1.00 | (0.68 |  | 1.47) | 0.9946 |
|  | Anti-hypertension | 2508 | 236 | 0.80 | (0.58 |  | 1.09) | 0.1508 |  | 0.80 | (0.58 |  | 1.09) | 0.1518 |
|  | Anti-platelet agent | 1398 | 122 | 0.68 | (0.53 |  | 0.88) | 0.0031 |  | 0.68 | (0.52 |  | 0.87) | 0.0028 |
|  | Amiodarone | 603 | 51 | 0.83 | (0.61 |  | 1.13) | 0.2442 |  | 0.83 | (0.61 |  | 1.12) | 0.2230 |
|  | Digoxin | 295 | 26 | 0.88 | (0.58 |  | 1.34) | 0.5424 |  | 0.87 | (0.57 |  | 1.33) | 0.5290 |
|  | Non-steroidal anti-inflammatory drug | 374 | 41 | 1.05 | (0.75 |  | 1.46) | 0.7962 |  | 1.05 | (0.75 |  | 1.47) | 0.7861 |
|  | Gastric antacids | 844 | 119 | 1.55 | (1.19 |  | 2.00) | 0.0009 |  | 1.53 | (1.18 |  | 1.99) | 0.0012 |

aHR=adjusted hazard ratio, CI=confidence interval

Supplemental Table 4. Cox proportional hazards regression model for pneumonia and hip fracture

| **Variable** | | **Outcome: pneumonia** | | | | |  | **Outcome: hip fracture** | | | | |
| --- | --- | --- | --- | --- | --- | --- | --- | --- | --- | --- | --- | --- |
|  |  | **aHR** | **95% CI** | | | ***p-value*** |  | **aHR** | **95% CI** | | | ***p-value*** |
| **Treatment** | |  |  |  |  |  |  |  |  |  |  |  |
|  | Warfarin | 1.00 |  | | |  |  | 1.00 |  | | |  |
|  | Apixaban | **0.99** | **(0.76** |  | **1.28)** | **0.9110** |  | **0.71** | **(0.29** |  | **1.70)** | **0.4363** |
| **Mean age (years)** | | 1.07 | (1.05 |  | 1.09) | <.0001 |  | 1.08 | (1.02 |  | 1.14) | 0.0081 |
| **Sex** | |  |  |  |  |  |  |  |  |  |  |  |
|  | Male | 1.10 | (0.87 |  | 1.40) | 0.4156 |  | 0.29 | (0.12 |  | 0.75) | 0.0104 |
|  | Female | 1.00 |  | | |  |  | 1.00 |  | | |  |
| **Baseline estimated glomerular filtration rate (ml/min/1.73 m^2^)** | | |  |  |  |  |  |  |  |  |  |  |
|  | ≧60 | 1.00 |  | | |  |  | 1.00 |  | | |  |
|  | 30-59.9 | 0.90 | (0.68 |  | 1.18) | 0.4353 |  | 1.13 | (0.46 |  | 2.82) | 0.7860 |
|  | <30 | 0.95 | (0.60 |  | 1.50) | 0.8358 |  | 2.46 | (0.56 |  | 10.70) | 0.2312 |
| **Baseline international normalized ratio** | |  |  |  |  |  |  |  |  |  |  |  |
|  | <1.5 | 1.00 |  | | |  |  | 1.00 |  | | |  |
|  | 1.5-2 | 1.39 | (0.98 |  | 1.98) | 0.0660 |  | 0.56 | (0.12 |  | 2.58) | 0.4556 |
|  | >2 | 1.18 | (0.82 |  | 1.68) | 0.3765 |  | 0.39 | (0.09 |  | 1.81) | 0.2316 |
|  | Missing | 0.72 | (0.47 |  | 1.11) | 0.1361 |  | 0.26 | (0.03 |  | 1.94) | 0.1870 |
| **Baseline comorbid condition** | | |  |  |  |  |  |  |  |  |  |  |
|  | Ischemic stroke/systemic embolism | 1.28 | (1.00 |  | 1.65) | 0.0519 |  | 1.69 | (0.72 |  | 3.99) | 0.2295 |
|  | Major bleeding | 1.32 | (1.02 |  | 1.70) | 0.0351 |  | 0.67 | (0.26 |  | 1.76) | 0.4211 |
|  | Hypertension | 0.84 | (0.63 |  | 1.13) | 0.2480 |  | 0.69 | (0.25 |  | 1.90) | 0.4710 |
|  | Renal disease | 1.72 | (1.26 |  | 2.36) | 0.0007 |  | 0.82 | (0.26 |  | 2.60) | 0.7308 |
|  | Liver diseases/severe liver diseases | 1.29 | (0.87 |  | 1.90) | 0.2114 |  | 0.56 | (0.07 |  | 4.35) | 0.5815 |
|  | Diabetes mellitus | 1.62 | (1.18 |  | 2.23) | 0.0029 |  | 1.83 | (0.56 |  | 5.93) | 0.3150 |
|  | Heart failure | 1.41 | (1.10 |  | 1.80) | 0.0060 |  | 1.18 | (0.48 |  | 2.93) | 0.7145 |
|  | Peripheral vascular disease | 1.73 | (1.09 |  | 2.77) | 0.0213 |  | 0.00 | (0.00 |  | . | 0.9863 |
| **Prior medication use** | |  |  |  |  |  |  |  |  |  |  |  |
|  | Lipid-lowering agent | 0.80 | (0.60 |  | 1.07) | 0.1270 |  | 0.54 | (0.20 |  | 1.45) | 0.2200 |
|  | Antidiabetic agents | 0.98 | (0.68 |  | 1.41) | 0.9042 |  | 0.93 | (0.25 |  | 3.40) | 0.9108 |
|  | Anti-hypertension | 0.80 | (0.59 |  | 1.10) | 0.1720 |  | 1.35 | (0.36 |  | 5.08) | 0.6554 |
|  | Anti-platelet agent | 0.73 | (0.57 |  | 0.95) | 0.0187 |  | 1.48 | (0.64 |  | 3.42) | 0.3618 |
|  | Amiodarone | 0.93 | (0.68 |  | 1.27) | 0.6605 |  | 1.60 | (0.66 |  | 3.85) | 0.2972 |
|  | Digoxin | 0.86 | (0.57 |  | 1.31) | 0.4829 |  | 0.97 | (0.26 |  | 3.70) | 0.9665 |
|  | Non-steroidal anti-inflammatory drugs | 1.07 | (0.77 |  | 1.50) | 0.6774 |  | 4.60 | (2.03 |  | 10.43) | 0.0003 |
|  | Gastric antacids | 1.49 | (1.15 |  | 1.94) | 0.0028 |  | 1.50 | (0.63 |  | 3.56) | 0.3617 |

aHR=adjusted hazard ratio, CI=confidence interval

Supplemental Table 5. Patient characteristics by apixaban dose

|  |  |  |  | **No. of patients** | **Reduced dose*, n=915** | |  | **Standard dose*, n=710** | | ***p-value*** |
| --- | --- | --- | --- | --- | --- | --- | --- | --- | --- | --- |
|  |  |  |  |  |  |  |  |  |  |  |
| **Age (years), n (%)** | | | |  |  |  |  |  |  | <.0001 |
|  | <40 | | | 12 | 2 | (0.22) |  | 10 | (1.41) |  |
|  | 40-64 | | | 245 | 69 | (7.54) |  | 176 | (24.79) |  |
|  | 65-74 | | | 504 | 217 | (23.72) |  | 287 | (40.42) |  |
|  | ≥75 | | | 864 | 627 | (68.52) |  | 237 | (33.38) |  |
| **Sex, n (%)** | | | |  |  |  |  |  |  | <.0001 |
|  | Male | |  | 938 | 484 | (52.90) |  | 454 | (63.94) |  |
|  | Female | | | 687 | 431 | (47.10) |  | 256 | (36.06) |  |
| **Baseline estimated glomerular filtration rate (ml/min/1.73m^2^), n (%)** | | | |  |  |  |  |  |  | <.0001 |
|  | ≥90 | | | 277 | 120 | (13.11) |  | 157 | (22.11) |  |
|  | 60-89.9 | | | 674 | 302 | (33.01) |  | 372 | (52.39) |  |
|  | 45-59.9 | | | 357 | 224 | (24.48) |  | 133 | (18.73) |  |
|  | 30-44.9 | | | 198 | 162 | (17.70) |  | 36 | (5.07) |  |
|  | 15-29.9 | | | 103 | 93 | (10.16) |  | 10 | (1.41) |  |
|  | <15 | | | 16 | 14 | (1.53) |  | 2 | (0.28) |  |
| **Baseline international normalized ratio, n (%)** | | | |  |  |  |  |  |  | 0.0085 |
|  |  | <1.5 | | 1291 | 744 | (81.31) |  | 547 | (77.04) |  |
|  |  | 1.5-2 | | 37 | 23 | (2.51) |  | 14 | (1.97) |  |
|  |  | >2 | | 17 | 13 | (1.42) |  | 4 | (0.56) |  |
|  |  | Missing | | 280 | 135 | (14.75) |  | 145 | (20.42) |  |
| **Mean (SD) value** | | | |  |  |  |  |  |  |  |
|  | Age at the index date, year | | | 1625 | 78.51 | (9.52) |  | 69.80 | (10.34) | <.0001 |
|  | Baseline eGFR, ml/min/1.73m^2^ | | | 1625 | 60.60 | (26.44) |  | 74.68 | (23.26) | <.0001 |
|  | Low-density lipoprotein, mg/dl | | | 1625 | 93.70 | (37.47) |  | 100.34 | (42.51) | 0.0032 |
|  | Congestive heart failure, Hypertension, Age ≥75 years, Diabetes, Stroke, Vascular disease, Age 65-74 years, Sex score | | | 1625 | 4.18 | (1.62) |  | 3.39 | (1.66) | <.0001 |
|  | Hypertension, Abnormal liver/renal, Bleeding, Labile INRs, Elderly (>65 years), Drugs (aspirin or NSAIDs) or alcohol score | | | 1625 | 3.13 | (1.32) |  | 2.66 | (1.33) | <.0001 |

*Standard dose of apixaban: 10 mg/day; reduced dose: 2.5-5 mg/day

Supplemental Figure 1. The mean international normalized ratios in patients treated with warfarin who developed systemic embolism (n=70)


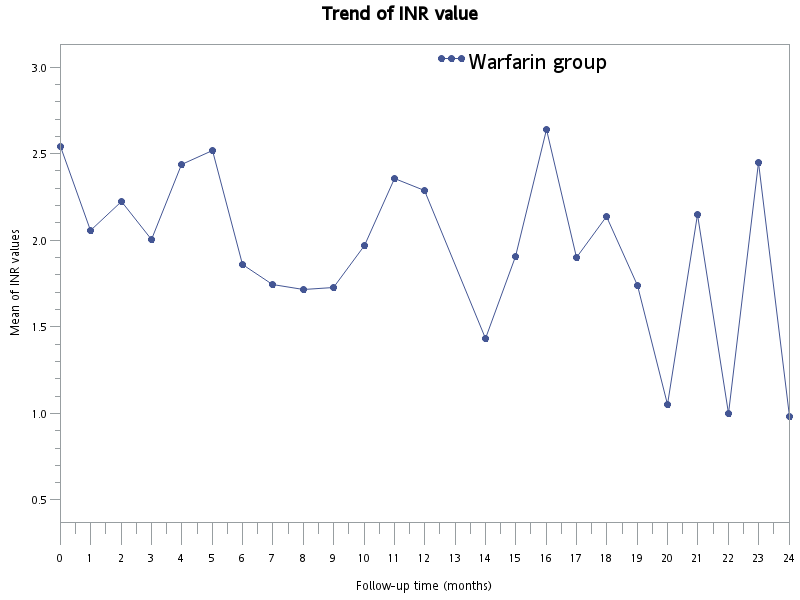


| Time (month) | Number of patient-test | Mean INR | SD |  | Time (month) | Number of patient-test | Mean INR | SD |
| --- | --- | --- | --- | --- | --- | --- | --- | --- |
| 0 | 85 | 2.54 | (1.52) |  | 13 | 4 | 3.14 | (1.67) |
| 1 | 90 | 2.05 | (1.11) |  | 14 | 9 | 1.43 | (0.51) |
| 2 | 54 | 2.22 | (1.13) |  | 15 | 7 | 1.91 | (0.60) |
| 3 | 36 | 2.01 | (0.90) |  | 16 | 16 | 2.64 | (2.00) |
| 4 | 40 | 2.44 | (1.15) |  | 17 | 3 | 1.90 | (1.14) |
| 5 | 44 | 2.52 | (1.51) |  | 18 | 12 | 2.14 | (0.78) |
| 6 | 22 | 1.86 | (0.59) |  | 19 | 3 | 1.74 | (0.79) |
| 7 | 14 | 1.75 | (0.59) |  | 20 | 1 | 1.05 | . |
| 8 | 29 | 1.72 | (0.72) |  | 21 | 1 | 2.15 | . |
| 9 | 20 | 1.73 | (0.76) |  | 22 | 1 | 1.00 | . |
| 10 | 16 | 1.97 | (0.85) |  | 23 | 1 | 2.45 | . |
| 11 | 5 | 2.36 | (1.31) |  | 24 | 1 | 0.98 | . |
| 12 | 8 | 2.29 | (1.26) |  |  |  |  |  |

Among the number of patient-tests for INR per month in the warfarin group, a high frequency of INR testing was within the first 5 months and most INR results were within the range of 2.0 and 2.5. Less frequent INR testing and great fluctuations (mean INR ranged, 1.72–3.14) occurred after the initial 5 months.

INR=international normalized ratio, TTR= time in therapeutic range (INR= 2.0-3.0)
